# Identification of the functional pathways altered by placental cell exposure to high glucose: lessons from the transcript and metabolite interactome

**DOI:** 10.1038/s41598-018-22535-y

**Published:** 2018-03-27

**Authors:** C. H. Hulme, A. Stevens, W. Dunn, A. E. P. Heazell, K. Hollywood, P. Begley, M. Westwood, J. E. Myers

**Affiliations:** 1 Maternal and Fetal Health Research Centre, Division of Developmental Biology & Medicine, School of Medical Sciences, University of Manchester, Manchester Academic Health Sciences Centre, Manchester, M13 9WL UK; 2 Maternal and Fetal Health Research Centre, Central Manchester University Hospitals NHS Foundation Trust, St Mary’s Hospital, Manchester Academic Health sciences Centre, Manchester, M13 9WL UK; 3 Division of Developmental Biology & Medicine, Faculty of Biology, Medicine & Health University of Manchester, Manchester Academic Health Sciences Centre, Manchester, M13 9WL UK; 4 Centre for Advanced Discovery and Experimental Therapeutics (CADET), Central Manchester University Hospitals NHS Foundation Trust, Manchester Academic Health Sciences Centre, Manchester, M13 9WL UK; 5 Centre for Endocrinology and Diabetes, Institute of Human Development, Faculty of Medical and Human Sciences, University of Manchester, Manchester, M13 9WL UK; 6 School of Biosciences, Phenome Centre Birmingham and Institute of Metabolism and Systems Research, University of Birmingham, Birmingham, B15 2TT UK; 7 Manchester Institute of Biotechnology and School of Chemistry, University of Manchester, 131 Princess Street, Manchester, M1 7DN UK

## Abstract

The specific consequences of hyperglycaemia on placental metabolism and function are incompletely understood but likely contribute to poor pregnancy outcomes associated with diabetes mellitus (DM). This study aimed to identify the functional biochemical pathways perturbed by placental exposure to high glucose levels through integrative analysis of the trophoblast transcriptome and metabolome. The human trophoblast cell line, BeWo, was cultured in 5 or 25 mM glucose, as a model of the placenta in DM. Transcriptomic analysis using microarrays, demonstrated 5632 differentially expressed gene transcripts (≥± 1.3 fold change (FC)) following exposure to high glucose. These genes were used to generate interactome models of transcript response using BioGRID (non-inferred network: 2500 nodes (genes) and 10541 protein-protein interactions). Ultra performance-liquid chromatography-mass spectrometry (MS) and gas chromatography-MS analysis of intracellular extracts and culture medium were used to assess the response of metabolite profiles to high glucose concentration. The interactions of altered genes and metabolites were assessed using the MetScape interactome database, resulting in an integrated model of systemic transcriptome (2969 genes) and metabolome (41 metabolites) response within placental cells exposed to high glucose. The functional pathways which demonstrated significant change in response to high glucose included fatty acid β-oxidation, phospholipid metabolism and phosphatidylinositol phosphate signalling.

## Introduction

Pregnancies complicated by diabetes mellitus (DM) are associated with poor maternal and perinatal outcomes. These include birth trauma^1^, stillbirth^2^ and pre-eclampsia^3^, however fetal overgrowth is the most common adverse outcome^4,5^. Infants with fetal macrosomia, diagnosed as those with a customised birth weight centile of 90 or greater, are more likely to develop metabolic syndrome in adulthood^6,7^. Whilst the association between maternal hyperglycaemia and excessive fetal growth is long established, the contribution of altered placental function to this relationship is incompletely understood^4^. Several studies have suggested that placental-fetal nutrient supply is altered in these pregnancies^8–13^. The molecular mechanisms that contribute to such dysfunction are poorly defined, although placental nutrient sensing pathways, such as the mammalian target of the rapamycin (mTOR) pathway^14,15^, and alterations in placental lipid metabolism^13,16^ have been implicated. The objective of the study presented here was to build on these observations regarding individual molecules and pathways by using a systems biology approach to obtain a holistic biochemical view of the placental response to high glucose.

Interactome networks that represent the transcript, metabolite and integrated transcript and metabolite response of a trophoblast cell line (BeWo) to culture in high glucose were generated. This method allows the visualisation and interpretation of complex interactions between large numbers of molecules^17^ and can therefore be used as a method of integrating multiple ‘omic datasets to provide an understanding of organisational complexity within the system^18^. Interactome networks are made up of nodes - the individual objects being studied, e.g., genes or metabolites - and edges - the connections between the objects, e.g., known protein-protein or protein-metabolite interactions^19^. Nodes that share large numbers of connections tend to share similar biological functions^19^. Therefore studying groups of proteins or proteins and metabolites that are highly interconnected, known as modules, can be used to identify key functions within an interactome network^19^. Conducting interactome network analysis alongside pathway ontology analysis, using tools such as Ingenuity Pathway Analysis (IPA)^20^, allows greater confidence in the selection of candidate pathways or molecules for further study as these are based on two independent methods of mapping the data, known protein-protein interactions and text mining, respectively.

Here we perform network and pathway analyses on transcript and metabolite data generated from an *in vitro* model of the placental trophoblast exposed to high glucose levels. These data reveal known, and importantly, novel functional pathways likely to be disrupted as a consequence of placental exposure to maternal hyperglycaemia.

## Materials and Methods

All reagents were purchased from Sigma-Aldrich unless stated.

### Cell Culture and Sample Preparation

BeWo cells (passage 10; n = 6; originally from the European Collection of Animal Cell Cultures, Porton Down; mycoplasma negative) were cultured on T75 and T225 flasks (Corning) (both seeded at 1.6 × 10^6^ cells/cm^2^), for transcriptomics and metabolomics, respectively. The number of replicates was based on similar numbers having been successfully used in other metabolomics studies where human samples were used, therefore less variability would exist in this cohort^21^. Cells were cultured for 24 hours in 1:1 DMEM:F12 containing 5 mM glucose and 10% fetal bovine serum (FBS), which was then exchanged for 1:1 DMEM:F12 containing either 5 mM (representing normoglycaemia) or 25 mM (representing hyperglycaemia;^22,23^) glucose and 10% FBS for a further 48 h. Preliminary studies were completed in which MTT assays were used to confirm that high glucose conditions (30 mM) did not affect cell viability compared to standard culture in 17 mM glucose (94 ± 34% (median ± IQR); n = 6; p > 0.05; Wilcoxon signed rank). Cells used for the analysis of RNA were lysed directly in Trizol® reagent (Invitrogen, UK), whereas those used for internal metabolome analysis were washed and quenched within 2.5 minutes of removing the conditioned medium (CM), scraped into suspension then subjected to 4 cycles of freezing with liquid nitrogen (60 s) and thawing on ice. CM was centrifuged (10,000 *g*; 10 min) and the supernatant was snap-frozen for analysis of the external metabolome. Cells and CM were stored at −80 °C until analysis.

### Microarray Analysis

Total RNA was isolated from the cell lysate using a Trizol® Plus RNA Purification Kit (Ambion, Paisley), according to the manufacturers’ instructions. RNA integrity and concentration was determined using a Nanodrop spectrophotometer and Agilent bioanalyzer. (Thermo Scientific, USA). Equal concentrations of RNA from each experimental replicate (n = 6) were pooled to an overall concentration of 95ng/μl and one microarray per experimental group was assessed.

The pooled samples were analysed using Affymetrix exon arrays (Affymetrix, High Wycombe, UK). Background correction, quantile normalization, gene expression analysis and robust multiarray analysis (RMA) of the data were completed in Bioconductor (Bolstad *et al*., 2003). Technical quality control and outlier analyses were performed using Affymetrix dChip software (Version 2005). Genes that had a fold change (FC) ≥  ± 1.3 between cells cultured in 25 mM compared to 5 mM glucose were identified for further analyses. Similar fold change cut-offs are commonly used for such network and pathway analysis approaches^24,25^. Partial least square discriminant analysis (PLS-DA) was applied using the MixOmics R-package^26,27^ and used to compare the fold changes of the selected genes to the unselected genes. Further assessment of specificity was performed by generating an affinity matrix from the gene expression data using the SNFtools R-package^28^ then t distributed stochastic neighbourhood embedding (tSNE) [Rtsne R-package^29^ was applied to show the clustering of genes with similar expression.

### Metabolomic Analysis

Two independent chromatography-mass spectrometry (MS) assays for metabolome analysis of the BeWo cells and conditioned CM were used to ensure that a wide range of polar and lipophilic metabolites were investigated. Full details of these methods are described in supplementary methods section A.

BeWo cells and CM were prepared as described previously^21,30^ (Supplementary Methods A1 and A2). Briefly, samples were lyophilised, then reconstituted in 50:50 Methanol:water for UPLC-MS analysis. Dried GC-MS samples were chemically derivatised *via* a process of methoxyimation then trimethylsilylation and then a retention marker solution was added. A quality control (QC) sample was prepared from a pool of all individual samples and for GC-MS analysis, succinic acid d_4_ was added as an internal standard to each sample.

Supplementary sections A4 and A5 detail complete methods for Ultra Performance Liquid-Chromatography MS. Samples were analysed in negative electrospray and positive ion modes on an Accela Ultra High Performance Liquid Chromatograph, coupled on-line to an electrospray LTQ-Orbitrap hybrid mass spectrometer (ThermoFisher Scientific, Hemel Hempstead, UK). The run order of samples was randomised. Data were processed using XCalibur (ThermoFisher Scientific, Bremen, Germany); applied in XCMS^31^ to assess relative quantification; chromatographic peaks were used to define the individual metabolic features and these features were matched according to accurate mass of metabolites by applying the software PUTMEDID_LCMS^32^. Putative (MSI level 2) and definitive identifications (MSI level 1) are reported.

Full details of the Gas Chromatography-Mass Spectrometry (GC-MS) analysis process are given in supplementary sections A6 and A7. Sample analysis was performed, within 24 h of derivatisation, using an Agilent 6890 gas chromatograph and 7673 autosampler (Agilent Technologies, Stockport, UK) attached to a LECO Pegasus III mass spectrometer (LECO Corporation, Stockport, UK). Pre-processing of GC-MS data was carried out, in which analyst-defined chromatographic peaks were associated with a retention index (RI) and electron impact (EI) mass spectrum for all samples and inputted into a study-specific peak list. Chromatographic peak deconvolution was performed for each sample and metabolite peaks were matched to peaks present in the study-specific list if defined criteria were met (RI ± 10, mass spectral (EI) match > 700). Peak areas were normalised to the succinic acid standard to generate a response ratio. Detected metabolite peaks were chemically identified by applying a search of the EI mass spectrum and RI in mass spectral libraries; the Golm metabolite library^33^, the National Institute for Standards in Technology database (NIST/EPA/NIH08 (NIST, 2010)), as well as over 500 entries in the MMD mass spectral/RI library^34^. Putative (MSI level 2) and definitive identifications (MSI level 1) are reported.

Multivariate analysis followed by Kruskal-Wallis testing of metabolite data were used to determine statistically significant differences (*p* < 0.01) in metabolite abundance between CM or cells cultured in 25 mM compared to 5 mM glucose. Only metabolites that could be assigned a PubChem ID, and that showed a differential abundance of ≥  ± 1.3 FC were used to analyse metabolite changes *via* pathway or network analysis.

### Pathway and network analysis of the transcriptome and metabolome

**Figure 1:**
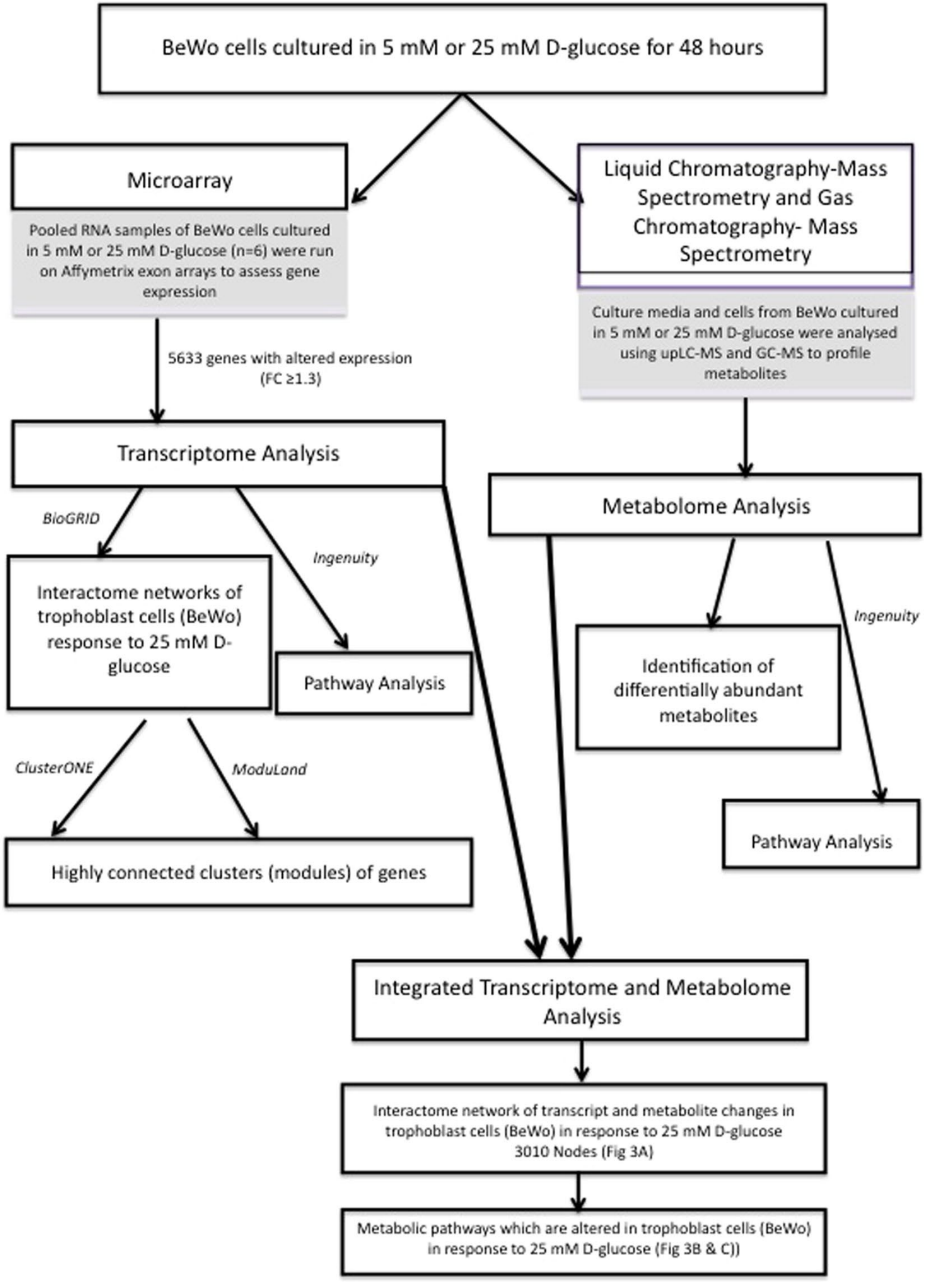
Overview of the workflow used to identify functional pathways which are altered within placental trophoblast cells in response to high glucose.

An overview of the approach taken is shown in Fig. 1. The key processes used to identify functional pathways altered in response to exposure of trophoblast to high glucose levels are detailed below.

### Network analysis of the transcriptome

The interactions between the differentially expressed genes were assessed using the BioGRID interactome database (v3.2.99) in Cytoscape (v2.8.3) to generate network models based on protein-protein interaction (either, only the genes identified from the array data (non-inferred nodes), or from the genes identified through the array analysis along with their inferred interactions (inferred nodes)^35^). Two independent mathematical algorithms, ClusterOne (v0.93)^36^ and Moduland (v2.8.3)^37^, were applied to the interactome networks in Cytoscape (v2.8.3) to identify highly connected clusters of proteins (modules) that are functionally central to the interactome network (ClusterOne) and that demarcate the hierarchical structure of the interactome network (Moduland). Modules identified using the ClusterOne algorithm were ranked based on their connectivity. Non-significant modules (*p* ≥ 0.05) were removed from further analysis. The biological function of each module was assessed by analysing the proteins identified within each cluster using the pathway analysis tool in Reactome software^38^. The significance of the pathway functions identified in Reactome was determined by Fisher’s exact test and *p* ≤ 0.05 was considered statistically significant.

### Pathway analysis

Genes that were differentially expressed and metabolites that were differentially abundant between cells exposed to 25 mM glucose compared to 5 mM glucose were analysed using pathway enrichment analysis (Ingenuity, Qiagen, US) to identify and visualize the affected canonical pathways. Pathways with *p* ≤ 0.05 were considered as statistically significant (Fisher’s exact test).

### Network analysis of a previously published transcriptome dataset of the murine placenta in a model of diabetes mellitus

We ensured that the functional pathways identified in this study were altered as a specific response of trophoblast, rather than just a choriocarcinoma cell line, to high glucose, by conducting a thorough NCBI PubMed search to identify published datasets of the placental transcriptome response to hyperglycaemia or diabetes mellitus (see full details in the supplementary methods section C). The data from a study that employed streptozotocin to induce DM in mice as a model of type 1 diabetes mellitus (T1 DM)^39^ were used to generate an inferred network from placental genes that were differentially expressed in the murine placental model of T1 DM compared to untreated mice ( ± 1.6 FC). Genes that overlapped between the BeWo analysis of 25 mM compared to 5 mM glucose and the mouse model of T1 DM, were then imported into a new inferred network. ClusterOne and Moduland algorithms were then applied to identify central gene clusters, as described above.

### Integrated network analysis of the transcriptome and metabolome

Differentially abundant metabolites (±1.3 FC) and genes (±1.3 FC) were analysed using the MetScape plugin (v2.0)^40^ in Cytoscape (v2.8.3) and networks generated based on known protein-protein and protein-metabolite interactions. The metabolic pathways which were associated with protein-metabolite interactions were mapped onto each of the networks to highlight the pathways with large numbers of gene and metabolite changes that were central within the network.

### Investigation of gene expression changes in an *ex vivo* placental explant model of high glucose and in placentas from women with T1DM

#### Collection and processing of placental samples and culture of term placental villous explants

Placentas were obtained with maternal informed, written consent in accordance with Local Research Ethics Committee approval (08/H1010/55, Manchester, UK). Placentas were collected within 30 minutes of delivery of a singleton infant at term (36 to 41 weeks gestation). Samples were taken from the centre, middle and edge of the placenta. Placental tissue was collected from women with T1 DM (n = 6) and BMI matched controls (BMI ≤ 30; n = 6). Patient demographics are shown in Supplementary Demographic Table 1. Gestation, birth weight and individualised birth centile (IBC) were also different across the groups, as women with T1DM, were delivered at approximately 36 weeks of gestation and gave birth to larger infants.

Placental explants were made as previously described^41^ from term placentas of uncomplicated pregnancies (Supplementary Demographic Table 2). Three placental explants were cultured per netwell in 1.8 ml of warmed CM (1:1 DMEM:F12), containing 5 mM DMEM:F12 and 10% FCS, overnight. CM was then replaced with 1.8 ml of either 5 mM or 25 mM D-glucose CM, containing 10% FBS for a further 48 hours.

All placental tissues, from explants or pregnancies complicated by DM were stored in RNAlater for later RNA extraction. Total RNA was extracted from placental explants and tissue using a Purelink RNA Mini kit (Ambion, Life Technologies) and quantified using a nanodrop (Nanodrop 2000c, ThermoScientific), according to manufacturer’s instructions. 250 ng RNA was used for reverse transcription (RT) and cDNA was generated as described previously^42^. RNA from the placental explant experiments, was pooled from 6 explants per experimental condition (each from separate netwells) for each of six placentas.

#### Quantitative real time- polymerase chain reaction (qRT-PCR) analysis of genes within the phosphatidylinositol phosphate pathway

qRT-PCR was used to corroborate the microarray data by assessing the effect of glucose on the expression of a subset of genes, choosing genes coding for proteins within the phosphatidylinositol phosphate pathway (AMP-Activated Kinase Alpha (AMPKα), Mammalian Target of Rapamycin (mTOR), P70 S6-Kinase (P70S6K) and 3-Phosphoinositide Dependent Protein Kinase 1 (PDK1)) as this pathway was identified by network and pathway analysis of the transcriptome, as well as the integrated transcriptome and metabolome to be functionally important in the BeWo cell response to high glucose. Further these genes were also assessed in samples derived from placental explants from uncomplicated pregnancies cultured in 5 mM or 25 mM glucose (Supplementary Methods B2 and B3) for 48 hours and placental tissue from women with and without T1DM (Supplementary Methods B4). Genes of interest were quantified using Brilliant III Ultra-Fast QPCR Master Mix (Agilent Technologies) on a MX3000 machine. qPCR reaction mixtures using standard (1×) primer concentration (0.25 μM) were made including the following primers: AMPKα F:ACCAGGTGATCAGCACTCCA, R:TCTCTTCAACCCGTCCATGC; mTOR F:TGTTCCGACGAATCTCAAAGC, R:TCATATGTTCCTGGCACAGCC; P70S6K F:GAGCTGGAGGAGGGGG, R:CCATGCAAGTTCATATGGTCC; PDK1 F:GGCCCAGAGTTGCTCAGAAT R: GCACTGGACTAACTGCCCAT. All samples were run in duplicate. 40 cycles of 95 °C for 3 minutes, 60 °C for 20 seconds and 72 °C for 30 seconds were performed. A standard curve was created from human reference RNA (1 µg/µl stock) ranging from 0.781 ng to 100 ng. Primer specificity was confirmed by analysis of dissociation curves generated within each run and by the inclusion of no RT and no cDNA controls’. Each of the genes was normalised to the mean of two reference genes, 18 S ribosomal RNA and Topoisomerase 1; both of which showed no difference in expression in response to 25 mM compared to 5 mM glucose.

## Results

### Gene changes in BeWo cells following culture in 25 mM compared to 5 mM glucose

The expression of 5632 gene transcripts, from the 133673 identified, differed ( ≥  ± 1.3 FC) between BeWo cells cultured in 25 mM compared to 5 mM glucose (Supplementary Table 1). PLS-DA analysis confirmed a significant difference (p < 0.001) between the genes with ≥  ± 1.3 fold change in expression and those that were altered to a lesser degree. Further analysis of the genes with ≥  ± 1.3 fold change in expression revealed 5 clusters, which map to pathways including lipid, (p value: 2.0 × 10^−6^ – 1.1 × 10^−2^), amino acid (p value: 1.0 × 10^−2^ – 1.6 × 10^−2^) and carbohydrate metabolism (p value: 1.5 × 10^−3^ – 7.6 × 10^−3^; Supplemental Fig. 1).

The 5632 genes defined as differentially expressed were used to generate two interactome networks using the BioGRID human interactome database. The first network contained only genes that were identified as altered by the microarray analysis (non-inferred network) and consisted of 2500 nodes (genes) and 10541 edges (protein-protein interactions). The second network was generated based on identified genes along with their inferred interacting partner genes (inferred network) and consisted of 10840 nodes and 59594 edges.

**Figure 2:**
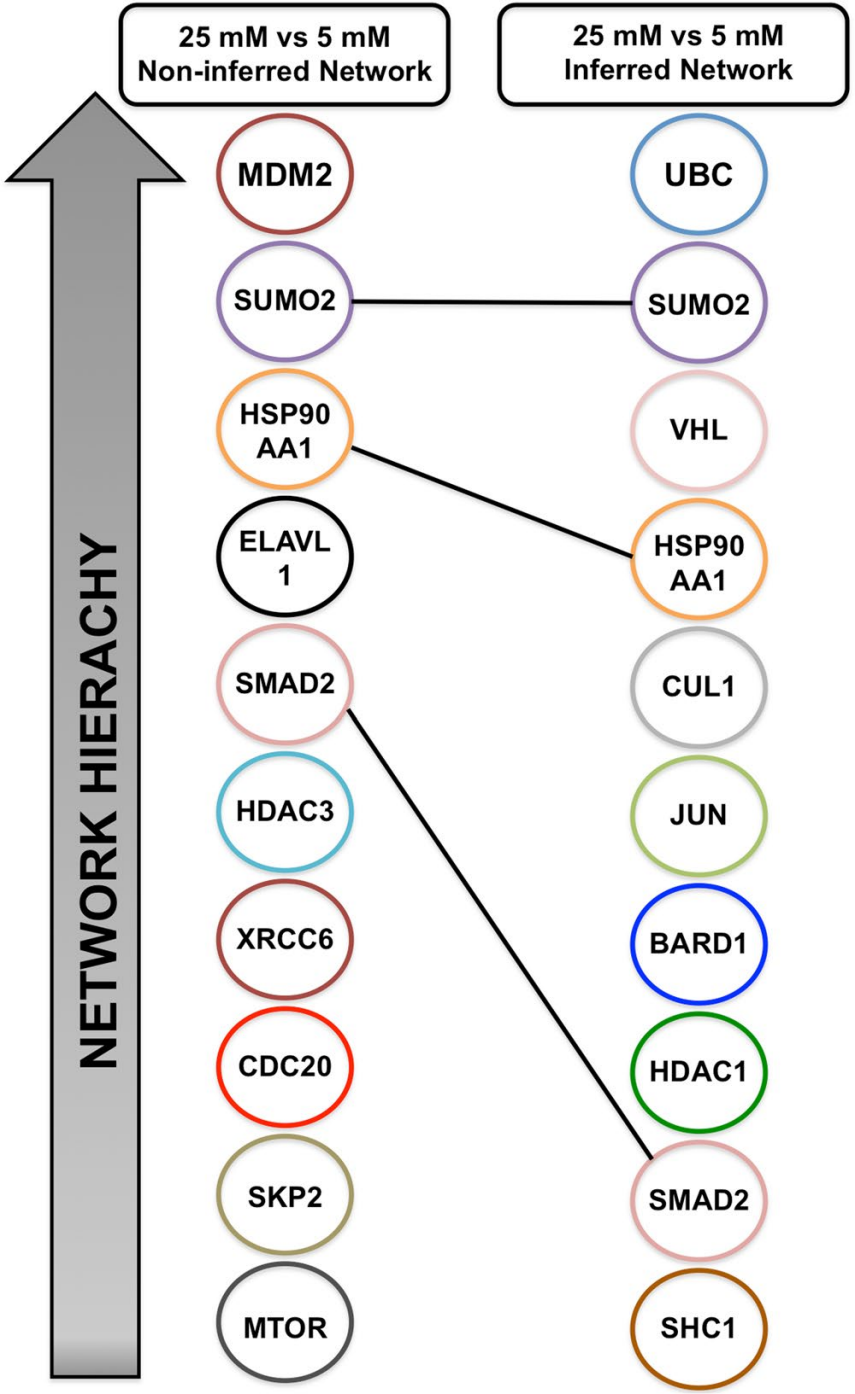
The ModuLand algorithm was applied to inferred and non-inferred interactome networks of gene changes (±1.3 FC) seen in BeWo cells cultured in 25 mM glucose compared to 5 mM glucose. Modules were identified from the network and are ranked based on their hierarchical network connectivity. Three modules were identified in both the inferred and non-inferred interactome networks.

Assessment of the networks hierarchy, using the Moduland algorithm, highlighted several modules (Supplementary Table 2); of the top ten (ranked by network centrality), three were common to the inferred and non-inferred networks (Fig. 2). The functions of the most hierarchically central modules (and the central protein associated with these modules/module name) within the non-inferred network were: phosphoinositide 3-kinase (PI3K) cascade (*MDM2; p* = 0.003), glucose metabolism (*SUMO2; p* = 0.004), peroxisomal lipid metabolism (*HSP90AA1; p* = 0.009), phospholipid metabolism (*ELAVL1*; *p* = 9 × 10^−4^) and signalling by Bone morphogenetic protein (BMP) (*SMAD2; p* = 3.9 × 10^−7^). Similarly, functions of the hierarchically central modules in the inferred network of transcript response to high glucose included: regulation of TP53 activity through acetylation (*SUMO2; p* = 1.49 × 10^−2^), cellular response to stress (*VHL; p* = 2.3 × 10^−13^), polyubiquitination of a substrate (*HSP90AA1; p* = 0.016), circadian clock (*CUL1; p* = 0.0003) and regulation of lipid metabolism by peroxisome proliferator-activated receptor alpha (*HDAC1; p* = *0.007*).

The ClusterOne algorithm generated 15 and 19 significant modules from the non-inferred and inferred interactome networks, respectively. The functions most significantly associated with the genes making up the most significant modules overlapped with those associated with the ModuLand-derived modules. Within the non-inferred network, these functions included: translation initiation (*p* = 2.32 × 10^−28^, Fisher’s exact test), glucose metabolism (*p* = 0.006), eIF2 activation (*p* = 0.002) and IGF1R signalling (*p* = 0.01). Modules identified within the inferred network (ClusterOne) were associated with lipoprotein metabolism (*p* = 0.004), insulin processing (*p* = 0.0005), circadian clock (*p* = 9 × 10^−4^) and peroxisomal lipid metabolism (*p* = 8.87 × 10^−13^). A summary of the modules identified using these algorithms and the functions associated with these modules are included in Supplementary Tables 2 and 3.

Pathway analysis, using the pathway enrichment tools in Ingenuity, suggested that the gene changes identified as a consequence of 25 mM compared to 5 mM glucose are likely to impact on numerous canonical pathways, many of which were confirmatory of the functional pathways associated with the interactome-derived modules. Altered functional pathways included, regulation of p70S6K signaling (p = 5.62 × 10^−7^), IGF-1 signaling (p = 4.51 × 10), insulin receptor signaling (p = 2.01 × 10^−9^) and mTOR signaling (p = 8.43 × 10^−4^).

#### Network analysis of a previously published transcriptome dataset of the murine placenta in a model of diabetes mellitus

80 genes overlapped between the murine model of T1 DM and the model of BeWo cells cultured in 25 mM glucose (Supplementary Table 6). An interactome network was generated which consisted of 1560 nodes and 1968 edges. Application of ClusterOne and ModuLand algorithms to the network identified 18 and 46 significant clusters, respectively. Functions of these clusters included regulation of lipid metabolism by peroxisome proliferator-activated receptor alpha (PPARα) and PI3K phosphorylation of phosphatidylinositol 4,5-bisphosphate (PIP2) to phosphatidylinositol (3,4,5)-trisphosphate (PIP3) (Supplementary Table 7). Full details of these results are included in the supplementary results section C.

#### Investigation of transcript changes of the phosphatidylinositol phosphate pathway using quantitative real time- polymerase chain reaction (qRT-PCR)

Table 1 demonstrates that altered expression of key genes within the phosphatidylinositol phosphate pathway, which were highlighted in the microarray analysis (AMPKα, mTOR, P70S6K and PDK1), could be confirmed using qRT-PCR. Assessment of expression of these genes in an independent sample set (n = 6) again demonstrated median FC differences with comparable levels of FC in the same direction of change as those from the microarray. Furthermore, the expression of these genes was assessed in an *ex vivo* explant model of high glucose, with three of the genes showing differential expression in the same direction as in the BeWo model. In the placentas (n = 6) from women with DM, AMPKα and P70S6K demonstrated differential expression in the same direction as the BeWo cells.

**Table 1:** Investigation of microarray data using qRT-PCR. RNA from six independent cultures of BeWo that had been pooled for analysis by microarray was analysed by qRT-PCR to determine the expression of a select panel of genes in order to investigate the microarray data. The median (IQR) fold change in gene expression observed in BeWo cells (n = 6) and placental explants (n = 6) cultured in 25 mM D-glucose compared to 5 mM D-glucose with the addition of 10% FCS is demonstrated; red = up-regulation and green = down-regulation. The fold change in gene expression that was observed in placental tissue from pregnancies complicated by type 1 diabetes mellitus (T1DM) compared to BMI-matched controls was calculated from the median expression values in each experimental group.

	25 mM D-glucose compared to 5 mM D-glucose (Fold Change)				T1 DM compared to Controls with a BMI ≤ 30 (n = 6)
	Trophoblast Cell model (BeWo): First experiment (pooled n = 6)		Trophoblast Cell model (BeWo): Second experiment (n = 6)	Explant Model (n = 6)	
	From Microarray	From qRT-PCR	From qRT-PCR	From qRT-PCR	From qRT-PCR
AMP-activated Protein Kinase Alpha (AMPK_α_)	−1.43	−1.79	−1.71 (1.48)	−1.21 (1.86)	−2.6
Mammalian Target of Rapamycin (mTOR)	+1.58	+1.57	+3.0 (3.00)	−1.06 (1.49)	+1.15
P70 S6-Kinase (P70S6K)	−1.15	−1.87	−3.0 (3.88)	−1.36 (1.95)	−1.61
3-Phosphoinositide Dependent Protein Kinase 1 (PDK1)	+1.43	+1.67	+1.14 (2.97)	+2.66 (2.79)	−1.08

### Metabolite changes in BeWo cells and their conditioned culture media following culture in 25 mM compared to 5 mM glucose

All experimental replicates were included in the metabolomic analyses. The effect of glucose on the metabolic footprint (conditioned CM) of BeWo cells was assessed using UPLC-MS and GC-MS. UPLC-MS analysis revealed that 51 metabolites were significantly different in the CM from BeWo cells exposed to 25 mM glucose (*p* ≤ 0.01) (Supplementary Table 4). The metabolites were categorised based on their class and the majority that were classifiable were found to be fatty acids and related metabolites (9 metabolites). GC-MS analysis identified only citrulline that was significantly increased (2.8 fold) in CM from BeWo cells cultured in 25 mM compared to 5 mM glucose (*p* = 0.004). BeWo cells (metabolic fingerprint) demonstrated 27 metabolites with significant differences between culture in 25 mM and 5 mM glucose (*p* ≤ 0.01), when assessed by UPLC-MS (Supplementary Table 5). GC-MS analysis identified 3 metabolites that were all increased in BeWo cells cultured in 25 mM compared to 5 mM glucose (Stearic acid, FC = 1.29, *p* = 0.02; Heptadecanoic acid, FC = 1.30, *p* = 0.03; Hexadecanoic acid, FC = 1.25, *p* = 0.05).

Pathway analysis of the metabolites within the BeWo cells and secreted culture medium suggested that the superpathways of Serine and Glycine Biosynthesis I (*p* = 3.71 × 10^−3^) and glycine biosynthesis I (*p* = 4.4 × 10^−3^) are altered as a result of exposure of BeWo cells to high glucose levels. The molecular and cellular functions likely to be altered included amino acid metabolism (*p* = 2.25 × 10^−11^) and small molecule biochemistry (*p* = 2.25 × 10^−11^).

### Interactome network analysis of the transcriptome and metabolome of BeWo cells following culture in 25 mM compared to 5 mM glucose

**Figure 3:**
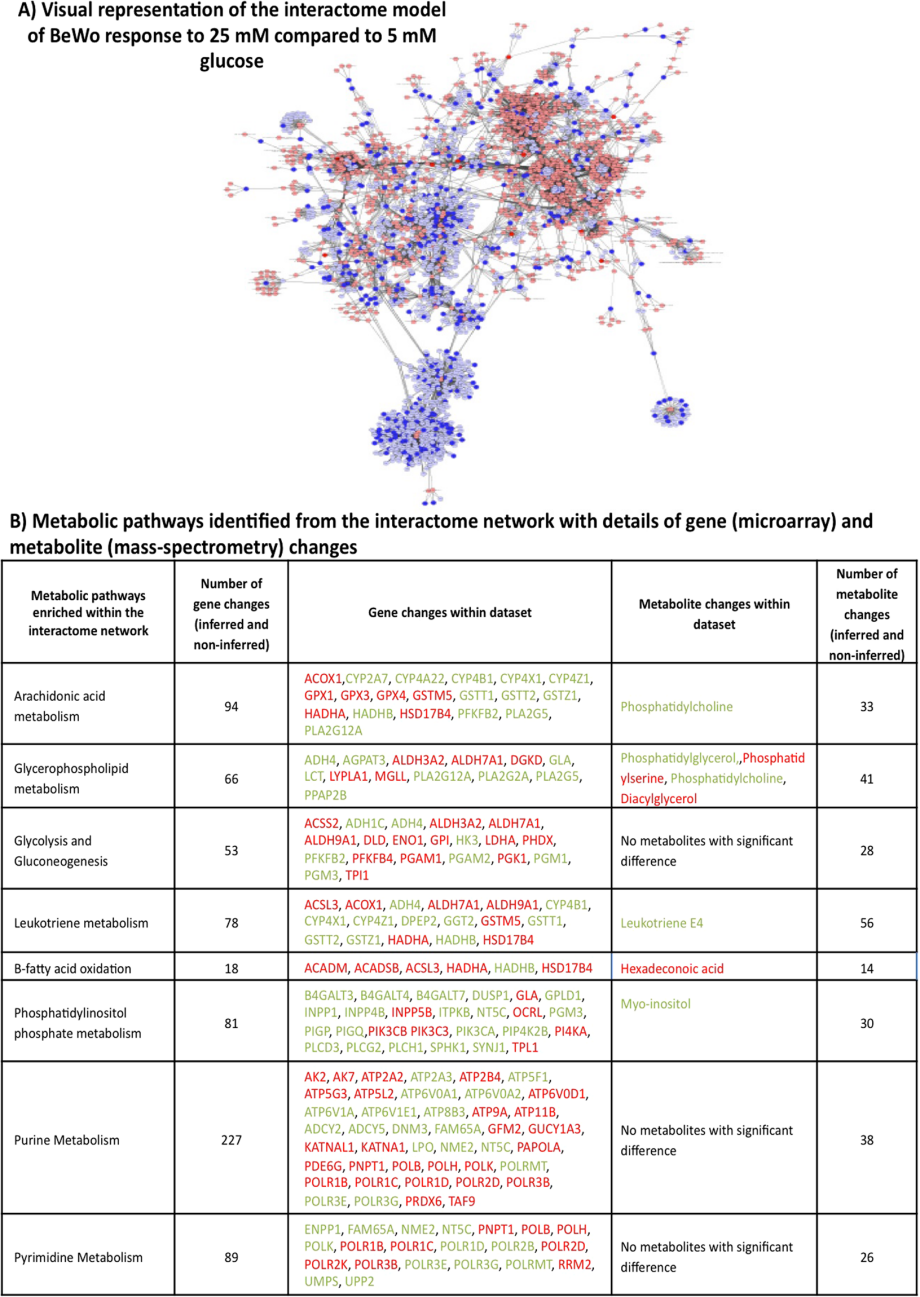
Network analysis of integrated gene and metabolite changes in BeWo cells cultured in 25 mM glucose compared to 5 mM glucose. (**A**) 5632 genes and 41 metabolites that were differentially expressed (±1.3 FC) in BeWo cells following 48 h culture in 25 mM compared to 5 mM glucose were used to derive an interaction network inferred using MetScape (3.1.1) as visually represented here; dark blue circles represent gene changes seen in the BeWo dataset, light blue circles represent inferred gene interactions, dark red circles represent metabolite changes seen in the BeWo dataset, light red circles represent inferred metabolite interactions, grey lines represent protein-protein or protein-metabolite interactions. (**B**) Table of the metabolic pathways with the greatest number of gene and metabolite changes that were identified from the integrated gene and metabolite interactome network. Genes or metabolites shown in red were up-regulated, whereas those in green were down-regulated.

An interactome network model (Fig. 3A) representing the integrated transcriptome and metabolome (intracellular and extracellular metabolites) response of BeWo cells to culture in 25 mM versus 5 mM glucose was generated which included 2969 of the differentially expressed genes and 41 of the differentially abundant metabolites that were connected via protein-protein or protein-metabolite interactions (Fig. 3). Analysis of this interactome network suggested that several biological functions are likely to be altered by changes to glucose concentrations. These modules included genes and/or metabolite interactions which were associated with purine metabolism, phosphatidylinositol phosphate metabolism and glycerophospholipid metabolism. The specific genes and metabolites within these modules are demonstrated in Fig. 3B and the differentially expressed/abundant genes and metabolites associated with these functional modules are highlighted in Fig. 3C.

## Discussion

The aim of this study was to identify transcripts and metabolites that were altered in trophoblast in response to high glucose and to then integrate these changes, using a systems-biology approach. Thus, ultimately aiming to characterise the molecular phenotype of the placental trophoblast in an interactome model, from which the functional pathways likely to be perturbed in placentas exposed to maternal hyperglycaemia could be identified. Although several candidate pathways were identified from individually analysing the transcriptome and metabolome data, interrogation of an integrated interactome model provides greater confidence that the pathways identified, which include some that have previously been associated with placental dysfunction in pregnancies complicated by DM as well as novel pathways, represent attractive candidates for future research relating to therapeutic interventions to prevent fetal overgrowth.

The phosphatidylinositol phosphate pathway (identified in transcriptome and integrated transcriptome and metabolome analyses), a key determinant of cellular proliferation and apoptosis^43^, is known to be regulated by hyperglycaemia in other organs^44,45^. Altered placental growth, particularly increased placental size/weight, has been widely demonstrated in pregnancies complicated by fetal macrosomia^46,47^; therefore this altered size could be associated with dysregulated placental proliferation due to perturbed PI3K pathway signalling. Moreover myo-inositol, the metabolite which forms the basis for this secondary messenger system^48^, has been implicated in several neonatal conditions in which fetal growth is atypical as researchers have reported increased levels in urine of neonates with fetal growth restriction (FGR)^49^ and decreased levels in FGR infants who go on to display catch-up growth^50^. Our investigations, to assess the expression of key genes within this pathway both corroborate the microarray data and suggest that expression of at least some of the genes within this pathway were similarly altered in an *ex vivo* placental explant model of high glucose and primary placental tissue from pregnancies complicated by DM as well as the BeWo trophoblast cell line. Together these observations suggest that this pathway warrants further investigation.

The three analyses of transcript, metabolite and integrated transcript/metabolite data have all indicated that trophoblast lipid metabolism is altered as a consequence of exposure to high glucose conditions. The placenta transports and metabolizes lipids essential for fetal development^51^ and it has been hypothesised that aberration in these functions may contribute to fetal macrosomia as excess lipid is supplied to the fetus, where it is stored within the fetal adipose tissue^52^. Some observations have supported this hypothesis in DM including: increased (39%) activity of placental lipoprotein lipase in insulin T1DM^13^ and decreased levels of β–fatty acid oxidation (FAO) in placentas of women with GDM^16^. Our study lends weight to this argument by suggesting that perturbed lipid metabolism, specifically β–FAO, may be a significant contributor to altered placental function in pregnancies complicated by DM directly as a consequence of hyperglycaemia.

Integration of our metabolome and transcriptome datasets proposed functional pathways not commonly associated with placental dysfunction in pregnancies complicated by DM, emphasising the potential of integrative network approaches for the identification of pathways for further study. One of these functional pathways, purine metabolism has not been studied in detail in the placenta, however, altered metabolism of the purine adenosine has been associated with increased nitric oxide synthesis in the placental macro- and micro-vascular endothelium^53^. In other pregnancy complications nitrative stress (caused by excess nitric oxide production^54^) is attributable to poor placental function^55,56^, therefore similar biochemical processes could be effected in placentas of pregnancies complicated by DM.

Our study is not only important in highlighting functional pathways within trophoblast that may be altered in response to high glucose, but it also demonstrates how these pathways interact to lead to systemic dysfunction. The integrated network provides a global representation of the subtle gene and metabolite changes which exist within the trophoblast cells following short-term exposure to high glucose. In a complex disease such as DM, it is likely that the phenotype is not due to changes in one pathway or an individual gene/metabolite, but attributable to a number of smaller changes which may interact with one another to lead to overall dysfunction of the biological network.

A major limitation of our study was that it relied on an *in vitro* trophoblast cell model of the placenta. The decision to use this model was driven by our ambitious aim to conduct a systems biology approach to generate and integrate large ‘omic datasets. Many of the studies which have been successful in utilising these approaches have done so using simple, highly controllable, single cell-type models^57^. Other studies have described the limitations of using the choriocarcinoma cell line, BeWo, as a model of trophoblast to investigate gene expression profiles as there are some disparities in basal gene expression when compared to primary trophoblast^58,59^. Encouragingly, we have determined that the expression of key genes within the phosphatidylinositol phosphate pathway are also altered in an *ex vivo* model of placental explant exposed to high glucose nonetheless, further studies are required to determine whether all of the candidate functional pathways identified in this study are similarly affected. In addition, the placenta contains a number of different cell types and therefore inclusion of whole placental tissue would have over complicated the analysis and significantly limited the interpretation of the data. Further analysis of a previously published transcriptomic dataset of the placenta in a murine model of T1 DM was included to ensure that the functional pathways identified in this study were altered as a specific response of trophoblast, rather than just a choriocarcinoma cell line, to high glucose. The functional pathways identified in the current study were highly conserved in the murine placental interactome network model, again adding greater confidence to the assertion that the BeWo interactome model described here is representative of the trophoblast response to high glucose.

It should also be recognised that this study has analysed transcriptome data generated from pooled RNA samples run on microarrays without technical replicates. Although not ideal for analysis of individual gene changes, significant changes were confirmed using PLS-LA and our analyses provided additional robustness by investigating how these genes contribute to a network of systemic by linking changes across the entire transcriptome. Moreover, these data have been integrated with metabolomic changes (including metabolites of known relevance to placental exposure to high glucose), thus adding a greater level of certainty that the interactome network generated in this study is likely to depict a model of the molecular phenotype of placenta in pregnancies complicated by hyperglycaemia. Furthermore, the expression of a panel of genes was determined by qRT-PCR analysis of the pooled samples used in the microarray, which supported the initial data and similar results were obtained when the same genes were assessed in a separate experiment, where samples from 6 independent replicates of BeWo cells exposed to high and low glucose for 48 h were analysed individually as well as in a pooled sample.

The work presented in this study is the first, to our knowledge, to investigate how trophoblast cells are altered by high glucose conditions using a systems biology approach. The interactome models generated in this study offer a unique insight into the complex interactions between placental genes and metabolites in response to high glucose and provides a platform for further *in vivo* or *ex vivo* studies to understand how the placenta responds to exposure to high glucose in pregnancies complicated by DM.

## Electronic supplementary material

Supplementary Material

Supplementary Dataset
