# Supplementary material for: Identification of the functional pathways altered by placental cell exposure to high glucose: lessons from the transcript and metabolite interactome
Source: Sci Rep. 2018 Mar 27;8:5270. doi: 10.1038/s41598-018-22535-y (PMC5869594; doi:10.1038/s41598-018-22535-y)
Supplement: Supplementary file 1 — Supplementary Material [file 41598_2018_22535_MOESM1_ESM.pdf]

Identification of the functional pathways altered by placental cell exposure to high glucose: lessons from the transcript and metabolite interactome

Authors: Hulme, C.H.[1,2], Stevens, A.[3], Dunn, W.[4,5,6], Heazell, A.E.P.[1,2], Hollywood, K.[4,5,7], Begley, P.[4,5], Westwood, M.[1,2] & Myers, J.E.[1,2]*

Institutions: [1]Maternal and Fetal Health Research Centre, Division of Developmental Biology & Medicine, School of Medical Sciences, University of Manchester, Manchester Academic Health Sciences Centre, Manchester, M13 9WL, UK; [2]Maternal and Fetal Health Research Centre, Central Manchester University Hospitals NHS Foundation Trust, St Mary's Hospital, Manchester Academic Health sciences Centre, Manchester, M13 9WL, UK; [3]Division of Developmental Biology & Medicine, Faculty of Biology, Medicine & Health University of Manchester, Manchester Academic Health Sciences Centre, Manchester, M13 9WL, UK; [4]Centre for Advanced Discovery and Experimental Therapeutics (CADET), Central Manchester University Hospitals NHS Foundation Trust, Manchester Academic Health Sciences Centre, Manchester, M13 9WL, UK; [5]Centre for Endocrinology and Diabetes, Institute of Human Development, Faculty of Medical and Human Sciences, University of Manchester, M13 9WL, UK; [6]School of Biosciences, Phenome Centre Birmingham and Institute of Metabolism and Systems Research, University of Birmingham, B15 2TT, UK; [7]Manchester Institute of Biotechnology and School of Chemistry, University of Manchester, 131 Princess Street, Manchester, M1 7DN, UK

Supplementary Materials

Supplementary Tables

Suppl. Table 1: Gene changes identified by microarray analysis. BeWo cells were cultured in 5 mM for 24 hrs before being switched to culture in either 5 mM or 25 mM glucose for 48 h (n=6). RNA was pooled from BeWo cultured in 5 mM or 25 mM and analysed using Affymetrix exon arrays. Background correction, quantile normalisation, robust multiarray analysis and gene expression analysis were performed in Bioconductor. The signal intensity of each transcript for each glucose condition is shown. The ratio and fold change in signal intensity of each transcript in BeWo cells cultured in 25 mM compared to 5 mM glucose is displayed.

Suppl.Table 2. Biological functions of modules generated using the ModuLand algorithm on interactome networks of BeWo cells response to culture in 25 mM compared to 5 mM glucose. The number of significant modules generated from both inferred and non-inferred interactome networks are shown. Genes from each of the significant modules were entered into Reactome, to identify possible biological functions associated with these genes. The biological function of the four modules with the most significant p value from each interactome network are shown.

Suppl. Table 3: Biological functions of modules generated using the ClusterOne algorithm on the interactome networks of BeWo cells response to culture in 25 mM compared to 5 mM glucose. The number of significant modules generated from both inferred and non-inferred interactome networks are shown. Genes from each of the significant modules were entered into Reactome, to identify possible biological functions associated with these genes. The biological function of the four modules with the most significant p value from each interactome network are shown.

Suppl. Table 4: Metabolites identified in the metabolic footprint (culture medium) of BeWo cells cultured in 25 mM compared to 5 mM glucose conditions using ultra performance liquid-chromatography mass spectrometry (UPLC-MS). BeWo cells (n=6) were cultured in 5 mM glucose overnight and then switched to 5 mM or 25 mM glucose conditions and culture media collected following a further 48 h. Samples were analysed using UPLC-MS and putatively named metabolite features that were significantly differently present in BeWo cell conditioned media following culture in 25 mM compared to 5 mM glucose are shown (Kruskal-Wallis; $p \leq 0.01$).

Suppl. Table 5: Metabolites identified in the metabolic fingerprint of BeWo cells cultured in 25 mM compared to 5 mM D-glucose conditions using ultra performance liquid-chromatography mass spectrometry (UPLC-MS). BeWo cells (n=6) were cultured in 5 mM glucose overnight and then switched to 5 mM or 25 mM glucose conditions and cells lysed following a further 48 h. Samples were analysed using UPLC-MS and putatively named metabolite features that were significantly differently present in BeWo cells cultured in 25 mM compared to 5 mM D-glucose are shown (Kruskal-Wallis; $p \leq 0.01$).

Supplementary Figures

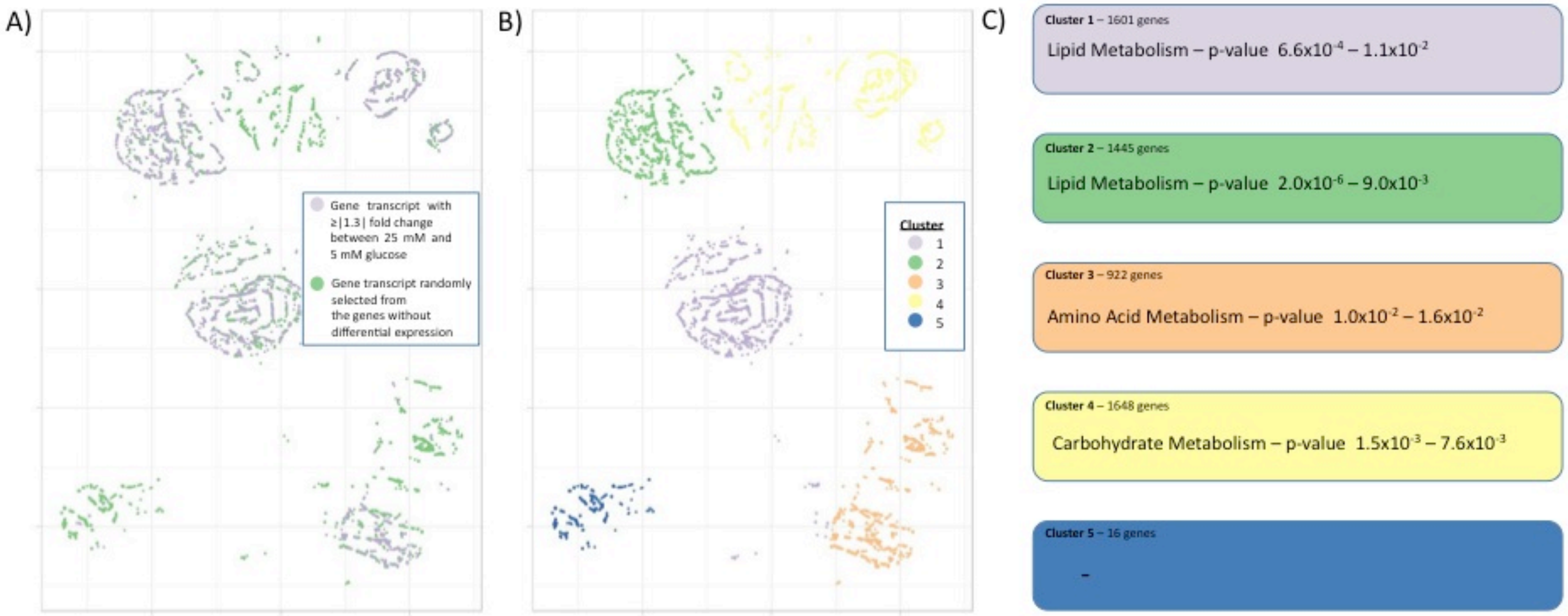

Supplemental figure 1. Analysis of genes which are differentially expressed between BeWo cells cultured in 25 mM compared to 5 mM glucose.
An affinity matrix was generated to assess the normalized similarity of differential gene expression between 25mM and 5mM glucose. Clusters of genes with similar differential expression were visualized using t-distributed stochastic neighborhood embedding (t-SNE). Axes are measures of the derived components with arbitrary scales. tSNE was performed using the Rtsne R-package.
A) Purple = 5632 gene transcripts with ≥|1.3| fold change between 25 mM and 5 mM glucose which were selected for further analysis, green = 5632 genes randomly selected from the genes without differential expression. The two groups localize to five gene clusters. **B)** Hierarchical clustering was used to define the five groups of genes, represented by different colours. **C)** Biological functions of the differentially expressed genes were determined using Ingenuity Pathway Analysis (IPA).

Supplementary Methods

A. Metabolomic analysis of BeWo cells and CM

The BeWo cells and CM samples were split 50:50 between UPLC-MS and GC-MS analyses.

A1 Metabolomic intracellular (fingerprint) sample preparation

3.5 mL of BeWo cell sample (extract in 70% Methanol) was transferred over 5 h to a 1.5 mL microcentrifuge tube (Eppendorf) whilst undergoing lyophilisation (Eppendorf Concentrator Plus, ambient temperature, ≈14h). Once dried, samples were reconstituted in 150 µL of 50:50 HPLC grade Methanol:HPLC grade water, centrifuged (13000 *g*; 15 min; ThermoScientific Heraeus Pico17), transferred to autosampler vials containing a fused 300 µL insert (ThermoFisher) and sealed with a silicone septa cap. A quality control (QC) sample was prepared by combining 50 µL aliquots of all samples analysed. 150 µL (for LC-MS) and 75 µL (for GC-MS) aliquots of the pooled QC sample were prepared and analysed as per biological samples. In GC-MS analysed samples, 50 µL of d^4 succinic acid was added as an internal standard to both the QC and biological samples.

A2 Metabolomic extracellular (footprint) sample preparation

CM samples underwent a protein precipitation step to remove any proteins; 600 µL MeOH was added to 200 µL sample and vortexed for 30 seconds, followed by centrifugation (13000; 15 min). 700 µL of sample was added to a 1.5 ml microcentrifuge tube (Eppendorf) followed by lyophilisation (Eppendorf Concentrator 120 Plus) for 5 h. Once dried, samples were reconstituted and QC samples prepared as for the metabolomic fingerprint. In GC-MS analysed samples, 50 µL of succinic acid was added as an internal standard to both the QC and biological samples.

A3 Chemical derivatisation for GC-MS analysis

Cell and conditioned CM samples being analysed by GC-MS were chemically derivatised in order to make them volatile enough to be eluted at reasonable GC temperatures [1]. Samples were chemically derivatised in a two-stage process. The first step involved methoxyimation (60 µL of 20 mg/mL of methoxylamine hydrochloride in dry pyridine (Acros Chemicals, UK) heated at 80°C for 20 min) and the second step involved trimethylsilylation (60 µL of N-methyl-N-(trimethylsilyl) trifluoroacetamide heated at 80°C for 20 min). Samples were allowed to cool, then 20 µL of a retention marker solution (n-alkanes C10, C12, C15, C19, C22, C26, C28, C30, C32, 3 mg/mL in dry pyridine) was added and

the samples were mixed by vortexing and then centrifuged (13000 *g*; 15 min; ThermoScientific Heraeus Pico17). 95 µL of each sample was transferred to an autosampler vial with a 300 µL fused insert and sealed with a silicone septa cap (ThermoFisher, UK).

A4 Ultra Performance Liquid Chromatography Mass Spectrometry (UPLC-MS) analysis

Samples were analysed on an Accela Ultra High Performance Liquid Chromatograph, coupled on-line to an electrospray LTQ-Orbitrap hybrid mass spectrometer (ThermoFisher Scientific, Hemel Hempstead, UK). A Hypersil GOLD column (100 x 2.1 mm, 1.9 µm; ThermoFisher Scientific, Runcorn, UK) with a column temperature of 50°C was used. The mass spectrometer was used in negative electrospray (ES-) and positive (ES+) ion modes separately, thus each sample was analysed twice, once in each ion mode. Two solvents were applied to the samples during the run in each ion mode. The solvents (A and B) contained 0.1% formic acid in water (vol/vol) and 0.1% formic acid in methanol (vol/vol), respectively. Both solvents were applied at a flow rate of 400 µL/min. Solvent A was held at 100% for 0.5 min followed by a linear increase to 100%; solvent B was then held at 100% for another 15.5 min. At 20.5 min a step change to 100% solvent A was performed and held for 1.5 min to equilibrate. Column eluent from the first 90 seconds was transferred to waste, after which it was transferred to the mass spectrometer.

The Orbitrap mass analyser (mass resolution 30,000 at *m/z* = 400) was applied to collect a full scan mass spectrum at each scan point in the mass range 50-1000. The following source and ion transfer parameters were used: source heater = 200°C, sheath gas = 50 (arbitrary units), capillary temperature = 300°C, aux gas = 15 (arbitrary units), automatic gain control (AGC) = $5x10^5$, ISpray voltage = 4kV (positive-ion mode) and 3kV (negative ion mode) and s lens =65%. Prior to running the samples, the mass spectrometer was mass calibrated following the manufacturers instructions. The run order of samples was randomised. Prior to the first sample injection, ten QC injections were analysed to make sure there was no run over of other samples within the MS. QC samples were then injected after every five sample injections, throughout the run.

A5 Processing of UPLC-MS data

NetCDF files, converted from the raw UPLC-MS data by the FileConverter programme available

in XCalibur (ThermoFisher Scientific, Bremen, Germany), were applied in XCMS [2] to construct a 3D data matrix composed of features (with associated accurate mass and retention time), sample ID and chromatographic peak areas. These data are reported as responses, calculated as chromatographic peak area. However, as calibration curves have not been created for each feature, these data can be used for relative quantification only. Following data processing, chromatographic peaks can be defined for the individual metabolic features. A single metabolite can be detected as many metabolic features. The chromatographic peaks are reported as accurate m/z, retention time and peak area. These metabolite features were then putatively annotated following the Metabolomics Standards Initiative guidelines [3] level 2, using Taverna to apply the PUTMEDID-LCMS identification workflow [4]. The accurate mass for each peak, of the putatively annotated metabolic features, was given either a single or multiple molecular formulas based on known formulas matching in mass to the experimentally determined mass with a mass error less than ±5 ppm. The molecular formula of individual features was matched to metabolites present in the Manchester Metabolomics Database (MMD) in order to identify specific metabolites [5]. Data were filtered further, based on expected retention time ranges, as single metabolites can be detected as multiple metabolic features, all with the same retention time but with different accurate masses [5]. Isomers and multiple features of the same metabolite can be classed as significantly different and are all reported.

A6 Gas Chromatography-Mass Spectrometry (GC-MS) analysis

Sample analysis was performed using an Agilent 6890 gas chromatograph and 7673 autosampler (Agilent Technologies, Stockport, UK) attached to a LECO Pegasus III mass spectrometer (LECO Corporation, Stockport, UK). Samples were randomly assigned an ID and a run order selected using Excel 2007. All samples were analysed within 24 h of the derivitisation process. Data were collected for four separate analytical batches.

Separations were carried out using a Varian VF-17MS column. Temperature was initially held at 70°C for 4 minutes, followed by a linear temperature ramp of 20°C per minute up to 300°C. 300°C was held for a further 4 minutes. The oven temperature was allowed to cool to 70°C prior to the next injection. The transfer line temperature was held at 240°C. The mass spectrometer source was operated at a temperature of 250°C in EI mode, with electron energy of 70 eV. Gas saver flow (25 ml min^{-1}) was switched on 15 seconds after sample injection. The detector operated in the range 1,400-1,800 V,

typically 50 V greater than the voltage determined during the LECO-defined tuning checks. Data were acquired over the range of *m/z* of 50-600, at an acquisition rate of 20 Hz.

A7 Data pre-processing for GC-MS data

Two samples from each metabolite class were chosen and chromatographic peak deconvolution was carried out using LECO ChromaTOF v4.22 software using the parameters: peak width 3 s, smoothing= 3. Analyst-defined chromatographic peaks were imported in to a study-specific database with associated retention index and electron impact mass spectrum for all samples. A number of metabolites were detected as multiple peaks as a result of different derivatization products being formed. Chromatographic peak deconvolution was performed for each study sample and metabolite peaks were matched to peaks present in the study-specific database if specific criteria were met (RI ± 10, mass spectral match > 700). The peak areas for all detected peaks were reported. Peak areas were normalised to a single internal standard (succinic d^4 acid)(peak area-metabolite/ peak area-internal standard) to generate a response ratio. Data were exported to Microsoft Excel 2007 version as ASCII files for data analysis.

Detected metabolite peaks were chemically identified by applying a search of the EI mass spectrum and retention index in mass spectral libraries; the Golm metabolite library [6], the national institute for standards in technology database, NIST/EPA/NIH08 [7], as well as over 500 entries in a mass spectral/RI library developed at The University of Manchester (MMD) [5]. Putative identifications (MSI level 2 or 3) were reported if the library and sample mass spectra had a match score greater than 700 in either the Golm or NIST/EPA/NIH08 metabolite libraries. Definitive identifications (MSI level 1) were reported if the library and sample mass spectra and retention index were matched with a match score greater than 700 and a retention index match +/- 10. Detected metabolite peaks were chemically identified by applying a search of the EI mass spectrum.

A8 Statistical analysis of UPLC-MS and GC-MS derived metabolites

Univariate analysis was used to determine whether relative concentrations of individual metabolites within the metabolic footprint and fingerprint differed between BeWo cultured in 25 Mm and 5 mM concentrations. A non-parametric Kruskal-Wallis test was used to ascertain which features were statistically different between samples cultured under different glucose conditions ($p < 0.01$).

B. Validation of gene expression changes in an *ex vivo* placental explant model and in placentas from women with T1DM

B1, Collection and processing of placental samples

Placentas were collected within 30 minutes of delivery of live infants by Caesarean section or vaginal delivery. All placentas were from term deliveries (36 to 41 weeks gestation). The mother's informed consent was gained according to Local Research Ethics Committee approval (Manchester, UK). The placenta was placed on a tray with the maternal side facing upwards. Full thickness tissue samples (1 cm^2) were cut from the centre, middle and edge of the placenta and washed twice in warm sterile PBS.

B2. Patient demographics of women with type 1 diabetes mellitus compared to uncomplicated pregnancy

Placental tissue was collected from women with T1 DM (n=6) and BMI matched controls (BMI ≤30; n=6). Patient demographics are shown in Supplementary Demographic Table 1. Gestation, birth weight and individualised birth centile (IBC) were also different across the groups, as women with T1DM, were delivered at approximately 36 weeks of gestation and gave birth to larger infants.

B3. Preparation of fresh term placental villous explants

Placental explants were made as previously described [8]. Placentas were collected and processed as in Supplementary methods B. Placentas were collected from uncomplicated pregnancies (Supplementary Demographic Table 1). The tissue was added to warm sterile PBS at 37°C and transferred to a biological safety cabinet; ensuring aseptic technique was maintained throughout tissue preparation.

The placental samples were washed three times with warmed PBS (37°C) to remove blood. Placental samples were then placed in sterile disposable Petri dishes (Corning Incorporated, US) with the maternal side facing upwards. Sterile scissors and forceps were used to remove the maternal decidua. 3 mm^3 pieces of placental villous tissue were dissected, using sterile scissors, to generate explants. Further blood and tissue debris was removed by washing in warmed, sterile PBS (37°C) and placed into a sterile Petri dish containing warmed CM, prior to culture

	T1 DM (n = 6)	Control (BMI ≤30) (n=6)	*p*
BMI (kg/m2)	24.7 (21 - 28)	22 (21 - 22)	*ns*
Maternal Age (years)	26 (19 - 37)	37 (33 - 39)	*ns*
Parity	0 (0-2)	1 (0 - 2)	*ns*
Caucasian (%)	80	80	*ns*
Smoker (%)	0	0	*ns*
Gestation (week+days)	36+6 (36+0 - 37+6)	39+0 (38+3 - 41+2)	$p >0.05$
Male (%)	80	60	*ns*
Vaginal Delivery (%)	0	0	*ns*
Caesarean Delivery (%)	100	100	*ns*
Birth Weight (g)	3560 (3240 - 4340)	3020 (2900 - 3360)	$p >0.05$
IBC	99 (55 - 100)	25 (12 - 65)	$p >0.01$

Supplementary Demographic Table 1: Demographic, obstetric and biophysical data for patient participants with type 1 diabetes mellitus (T1 DM), type 2 diabetes mellitus (T2 DM) and BMI matched controls. Data are median (range). Abbreviations: BMI, body mass index; IBC, individualised birth weight centile. *P*-values were calculated for a) T1 DM compared to controls with a BMI ≤30 and b) T2 DM compared to controls with a BMI ≥30 using Kruskal-Wallis with Dunn's post-hoc. Non-significant differences are represented as *ns.*

B3. Culture of placental explants

Placental explants were transferred into 12 well culture plates (Corning Incorporated) containing sterile netwells (Corning Incorporated) in 1.8 ml of warmed CM (1:1 DMEM:F12), containing 5 mM DMEM:F12 and 10% FCS. Three explants were cultured in each netwell. Explants were maintained at 37°C in 5% CO2. Following overnight culture, CM was removed from the wells using an aspirator and replaced with 1.8 ml of either 5 mM or 25 mM D-glucose CM, containing 10% FBS for a further 48 hours, replenishing explants with fresh CM after 24 hours (n=6).

C. Investigating the effect of type 1 diabetes mellitus on the murine placental transcriptome

An NCBI GenBank search of the Gene Expression Omnibus (GEO) was carried out to identify transcriptomic datasets of placental gene change from a mouse model of DM. The keywords: diabetes, hyperglycemia, hyperglycaemia, placenta, high glucose and trophoblast, were used for this search (completed in May, 2013). One study was identified, in which streptozotocin was used to induce DM in mice as a model of T1 DM [9]. The accession number for this study is GE 28277. The data from this study were analysed with QluCore Omics explorer (v2.3) to identify genes which were significantly altered in placentas from the mouse model of DM compared to control mice.

An inferred network was generated from placental genes that were differentially expressed in the murine placental model of T1 DM compared to untreated mice (±1.6 FC). A fold change cut-off of ±1.6 was used as this was the cut-off used in the original study [9]. The total genes generated from the inferred network were converted to human orthologs using Ingenuity. Genes were plotted into Venn diagrams using Venny online software (www.bioinfogp.cnb.csic.es/tools/venny/index.html), and compared with the genes generated from the inferred network of the BeWo response to 25 mM compared 5 mM glucose.

	Normal Pregnancy (n=6)
BMI (kg/m2)	23.8 (21 - 27)
Maternal Age (years)	34.5 (29 - 38)
Parity	1.5 (0 - 2)
Caucasian (%)	50
Smoker (%)	0
Gestation (week+days)	39+2 (38+2 - 40+5)
Male Infant (%)	50
Birth Weight (g)	3408 (2850 - 3852)
IBC	48 (12 - 74)
Vaginal Delivery (%)	17
Caesarean Delivery (%)	83

Supplementary Demographic Table 2: Demographic, obstetric and biophysical data for patient participants used for placental explants. Data are median (range). Abbreviations: BMI, body mass index; IBC, individualised birth weight centile.

Genes that overlapped between the BeWo analysis and the mouse model of T1 DM, were then imported into a new inferred network and ClusterOne and Moduland algorithms applied to identify central gene clusters, as described in the main text methods.

Supplementary Results

C. Investigating the effect of type 1 diabetes mellitus on the murine placental transcriptome

One of the limitations of our BeWo cell model is that it is based on analysing data from an *in vitro* cell line model of trophoblast. As transcriptome analysis has become a widely used tool throughout the scientific field, we aimed to utilise previously published transcriptomic datasets from an *in vivo* model of trophoblast exposed to high glucose to validate our *in vitro* model dataset, using a network biology approach.

C1. Identifying an Appropriate Study

We aimed to find a study that had performed microarray analysis of RNA from placental tissue exposed to high glucose or DM *in vivo*. An NCBI GenBank search was carried out to identify datasets that would be suitable for this purpose.

Only one study in which an appropriate experimental design had been carried out was identified [9]. The transcriptomic dataset was generated from placental tissue of mice that had a diabetic phenotype induced using steptozotocin (100 mg/kg body weight). Placentas were collected at embryonic day 10.5 and RNA samples run on individual arrays (Affymetrix mouse 430 2.0) for each sample: model of DM (n=5) and controls (n=5). Primary data files were available from the NCBI Gene Expression Omnibus Repository (accession number GE 28277). This study found 158 genes that were differentially expressed (±1.6 FC) in placentas from the mouse model of DM compared to control mice.

C2. Identifying gene changes that overlap between a murine model of T1 DM and a placental trophoblast model of high glucose

Genes which were changed in the BeWo cell model of high glucose were plotted, along with genes which changed in the mouse model (± 1.6 FC) into Venn diagrams using Venny. Genes present in the two models were used to generate a new interactome network, using Cytoscape (v2.8.3). ClusterOne and ModuLand algorithms were then applied to each of the networks, to identify clusters, central to the networks function, which could then be assigned biological functions using Reactome.

80 genes overlapped between the murine model of T1 DM and the model of BeWo cells cultured in 25 mM D-glucose (Supplementary Table 6). An interactome network was generated which consisted of 1560 nodes and 1968 edges. Application of ClusterOne and ModuLand (round 1) algorithms to the network identified 18 and 46 significant clusters, respectively. Functions of these clusters included regulation of lipid metabolism by peroxisome proliferator-activated receptor alpha (PPARα) and PI3K phosphorylation of phosphatidylinositol 4,5-bisphosphate (PIP2) to phosphatidylinositol (3,4,5)-trisphosphate (PIP3) (Supplementary Table 7).

Supplementary References

1. Orata F. Derivatization reactions and reagents for gas chromatography analysis. In: Advanced Gas Chromatography – Progress in Agricultural, Biomedical and Industrial Applications. 83–156 (2012).

2. Smith CA, Want EJ, O'Maille G, Abagyan R, Siuzdak G. XCMS: processing mass spectrometry data for metabolite profiling using nonlinear peak alignment, matching, and identification. *Anal Chem.* **78**, 779–87 (2006).

3. Sumner LW, A. A, Barrett D, Beale MH, Beger R, Daykin CA, et al. Proposed minimum reporting standards for chemical analysis Chemical Analysis Working Group (CAWG) Metabolomics Standards Initiative (MSI). *Metabolomics*, **3**, 211–21 (2007.

4. Brown M, Wedge DC, Goodacre R, Kell DB, Baker PN, Kenny LC, et al. Automated Workflows for Accurate Mass-based Putative Metabolite Identification in LC/MS-derived Metabolomic Datasets.

Bioinformatics. **27**, 1108–12 (2011).

5. Brown M, Dunn WB, Dobson P, Patel Y, Winder CL, Francis-McIntyre S, et al. Mass spectrometry tools and metabolite-specific databases for molecular identification in metabolomics. *Analyst*. **134**, 1322–32 (2009).

6. Golm Library. Golm Library. 2010. http://csbdb.mpimgolm.mpg.de/csbdb/gmd/msri/gmd_contributions.-html.

7. NIST. NIST Standard Reference Database. NIST/EPA/NIH08. 2010.

8. Simán, C.M., Sibley, C.P., Jones, C.J., Turner, M.A. & Greenwood, S.L. The functional regeneration of syncytiotrophoblast in cultured explants of term placenta. *American journal of physiology. Regulatory, integrative and comparative physiology*. **280,** R1116– R1122 (2001).

9. Salbaum, J.M., Kruger, C., Zhang, X., Delahaye, N.A., Pavlinkova, G. et al. (2011). Altered gene expression and spongiotrophoblast differentiation in placenta from a mouse model of diabetes in pregnancy. *Diabetologia*. **54**, 1909–1920 (2011)
